# A Latent Class Analysis of Gender Attitudes and Their Associations with Intimate Partner Violence and Mental Health in the Democratic Republic of Congo

**DOI:** 10.3390/ijerph18084063

**Published:** 2021-04-12

**Authors:** Andrew Corley, Nancy Glass, Mitima Mpanano Remy, Nancy Perrin

**Affiliations:** 1 School of Nursing, Johns Hopkins University, 525 North Wolfe Street, Baltimore, MD 21205, USA; nglass1@jhu.edu (N.G.); nperrin@jhu.edu (N.P.); 2 Programme d’Appui aux Initiatives de Développement Economique au Kivu (PAIDEK), Bukavu, Democratic Republic of Congo; mitimaremy@yahoo.fr

**Keywords:** gender, intimate partner violence, mental health, Sub-Saharan Africa

## Abstract

Gender role attitudes, views held by individuals regarding the roles men and women should play in society, are a powerful social determinant of health. However, work remains in elucidating the associations between gender attitudes and intimate partner violence (IPV) perpetration or victimization and mental health problems. We used latent class analysis to classify patterns of responses on survey items on gender attitudes by male and female adults in households that participated in an economic empowerment intervention and evaluation in rural villages in the Democratic Republic of Congo. Attitudes about IPV and gender equality were two subdomains to emerge from analysis and a 3-class model solution was found to best fit response patterns. Results indicated that, as compared to the least gender equitable class, individuals in the moderately gender equitable and fully gender equitable classes had lower odds of having experienced or perpetrated psychological abuse. Individuals within the moderately gender equitable class were at lower odds of having experienced or perpetrated physical or sexual violence. Further, individuals in the moderately gender equitable and fully gender equitable classes had significantly lower mean scores on symptoms associated with PTSD than individuals in the least gender equitable class. Future research should explore the relationships between gender attitudes, partner violence and mental health to build resilient families.

## 1. Introduction

Inequitable gender attitudes result from differences in views held by individuals regarding the roles men and women should play in society and are an important social determinant of health [1,2,3]. For women, beginning at a young age inequitable gender attitudes frequently restrict independence, educational opportunities, and the roles that they may occupy in public life. This narrowed scope of possibilities combines with beliefs about the importance of female subservience, leading to fewer economic opportunities; increased risks for early marriage and violence in the relationship; and reduced mental and physical health [4,5]. For men, inequitable gender attitudes are the foundation for notions of masculinity that favor toughness and dominance over other personality characteristics considered less socially valuable [4]. Such hegemonic masculinities can be damaging to individuals’ and families’ health and well-being by predisposing men to violence and substance abuse; discouraging their participation in caregiving and household chores; promoting risky sexual behaviors; and permitting intimate partner violence [6,7,8].

The different social roles and expectations of men and women in a society that simultaneously shape and are reinforced by gender inequitable attitudes often create unequal power dynamics in which one gender is empowered to subordinate the other [9]. Often, the maintenance of these power dynamics is achieved by acts of violence by men against women. Gender-based violence (GBV), violence against women, is a global public health threat and a consequence of gender inequitable norms [4]. Intimate partner violence (IPV)—emotional, verbal, economic, physical or sexual abuse perpetrated by a domestic partner—is estimated to affect one in three ever-partnered women worldwide, making it one of the most frequently reported forms of GBV [10]. IPV oftentimes leads to negative mental health outcomes, including post-traumatic stress disorder, anxiety, depression, low self-esteem, suicidality, and substance abuse [11].

Despite the existence of dominant gender attitudes within a society, community member attitudes that are informed by and reinforce them are not monolithic [12]. Within a community, individuals may express an array of different attitudes toward gender and gender roles. Such attitudes are learned through a process of socialization that occurs within families, between peers, at workplaces, and through interactions with individuals and institutions at every level of one’s social ecological environment [13,14]. Exposure to armed conflict and the widespread use of physical and sexual violence against both women and men is a critical life event that can profoundly impact both individuals’ gender attitudes and the psychological health of whole communities [15]. While research suggests that gender inequality and armed conflict reciprocally exacerbate one another [16], it remains necessary to further explore the lasting impact of living in a physically and economically insecure post-conflict setting on attitudes toward gender and IPV and their associations with mental health. The importance of conducting such research becomes increasingly clear as scholarship continues to clarify how gender attitudes and emotional trauma are transmitted intergenerationally to influence the health of future generations [17,18,19]. In order to help meet this gap in research, this study will draw upon data from economic empowerment studies conducted in the eastern Democratic Republic of the Congo (DRC), an area that has experienced nearly three decades of insecurity and widespread reports of sexual and gender-based violence [20]. While its post-colonial history has been marked by numerous periods of violence, many of the DRC’s current-day conflicts have roots can be traced back to the 1994 Rwandan Genocide and Congo Wars. Since the end of these largescale conflicts, the eastern DRC has been site of a series of armed conflicts between non-state and state actors [20].

The objectives of this study are to classify men and women in households living in a post-conflict setting based on their responses to a survey on gender attitudes and to examine associations between these classes and their demographic characteristics, histories of IPV victimization or perpetration, and mental health outcomes. To accomplish this, we use latent class analysis (LCA), a model-based clustering approach, to classify people into distinct patterns of gender attitudes based on responses to items on a gender equality survey. Creating this classification structure provides the opportunity to estimate the proportion of the overall sample that may share similar gender attitudes and to unearth the associations such attitudes have with demographic characteristics, past histories with experiencing or perpetrating IPV, and current mental health problems. We hypothesize that those classes characterized by more restrictive gender attitudes will be associated with greater odds of having experienced or perpetrated IPV and worse mean scores on measures of current mental health problems.

## 2. Materials and Methods

### 2.1. Parent Study

Data for this analysis comes from cross-sectional survey data of adult participants (*n* = 784) enrolled in a related series of randomized control trials whose aims were to improve a number of outcomes related to economic well-being, family functioning, and gender equality. The first study, Pigs for Peace (PfP), evaluated the effectiveness of a livestock asset transfer program for adults living in ten rural villages in South Kivu, DRC [21]. Within the study, each adult participant randomized to receive the intervention received a female piglet, care instructions, and ongoing technical support by the local non-governmental organization (NGO) implementing partner. In exchange for the piglet, participants agreed to repay the local NGO with the first two piglets it birthed and these repayment piglets served as asset loans for other households in the program. After meeting this program requirement, participants were mentored to continue the livestock project as an ongoing income source to meet basic needs. The second study compared the relative effectiveness of PfP with that of a second animal/livestock asset transfer program targeting adolescents aged 10 to 15 years titled Rabbits for Resilience (RfR). In a similar scheme, adolescents and their families were randomized to one of three arms (rabbit only, pig only, rabbit and pig) and technical support from the local NGO in exchange for agreeing to repay the first two offspring of the animal/livestock provided to the household to the partner NGO [22]. The PfP and RfR interventions’ effectiveness in improving adolescent well-being outcomes were compared after a 24 month trial period. All survey data was translated into French and administered verbally in participants’ preferred language (French, Mashi, or Swahili) by experienced Congolese research assistants. Responses were recorded in French and then translated into English before analyses.

### 2.2. Measures

#### 2.2.1. Attitudes towards Gender Equality

Gender attitudes were collected using an adapted version of the Social Norms and Beliefs about Gender-Based Violence Scale, a 30-item scale designed to measure changes over time in personal beliefs and social norms thought to maintain tolerance of sexual violence and related forms of gender-based violence in low-resource and complex humanitarian settings [23]. As the present study’s objective was to classify individuals in the parent study based on their gender equality attitudes rather than attitudes specific to sexual violence, we included only the items on the scale that related to the construct of gender equality. We adopted the Inter-Agency Standing Committee’s (IASC) definition of gender equality as our guiding theoretical construct. The IASC states that gender equality is the “equal enjoyment of women, girls, men, and boys—of all age, sexual orientations and gender identities—of rights, goods, opportunities, resources, rewards and quality of life” [24]. In order to operationalize this definition, we selected scale items that aligned with targets of Goal 5 of the Sustainable Development Goals (SDGs): achieving gender equality and empowering all women and girls that were culturally relevant to the study population and viewed as influencing partner violence and mental health [25]. Following discussion between the three authors, eight scale items were retained for inclusion in the latent class analysis. These items were then arranged to reflect the two subdomains: “husbands’ use of intimate partner violence” and “gender equality.” In order to conduct the analysis, it was necessary to dichotomize the four-point Likert scales of each item. Scales were divided in half and the lower two possible responses of the original scale expressing disagreement with an item’s gender equitable attitude were rescored as 0. The remaining two possible responses expressing agreement with this attitude were rescored as 1. A score of 0 represented disagreement with an item’s statement and 1 represented agreement.

#### 2.2.2. Demographic Characteristics

Participants’ age, sex, education, marital status, and food insecurity were collected. Food insecurity and relative socioeconomic status were captured using the Household Food Insecurity Access Scale (HFIAS), an experience-based food insecurity scale covering a 30 day recall period [26]. In previous research, the HFIAS has been shown to be strongly related to socioeconomic status, indicating improved food access with higher socioeconomic status [27,28]. The nine scale items were used to measure the frequency of occurrence of behavioral and psychological manifestations of food insecurity using a 4-point Likert scale from 0 “never” to 3 “often” with higher scores representing greater food insecurity. Mean scores were then calculated for each participant.

#### 2.2.3. IPV Experiences

Female participants were also asked whether they had ever experienced controlling behaviors or psychological abuse and/or, physical, and/or sexual violence by their male partners. Male participants were asked about their perpetration of any of these IPV behaviors against their female partners. Data was collected using an 18-item measurement tool with a dichotomous yes/no scale for each item. Items asked about experiences of (1) jealous; (2) accuse of being unfaithful; (3) does not allow you to see friends; (4) limits contact with your family; (5) insists on knowing where you are; (6) does not trust you with money; (7) humiliate; (8) threaten to hurt; (9) insult; (10) push; (11) slap; (12) twist arm or pull hair; (13) punch; (14) kick, drag, or beat; (15) choke or burn; (16) threaten or attack with a weapon; (17) force to have sexual intercourse; and (18) force to perform sexual acts. Items 1 through 6 measured controlling behavior, items 7 to 9 correspond to psychological abuse, items 10 through 18 correspond to physical violence, and items 11 and 12 correspond to sexual violence. A participant reported IPV experienced or perpetrated if they responded affirmatively to any of its corresponding questions. For the purposes of this analysis, physical and sexual violence were combined into a single form of IPV due to the relative rarity of reported sexual violence.

#### 2.2.4. Mental Health

Participants were asked to describe any recent or ongoing feelings of anxiety or depression. The Hopkins Symptom Checklist-25 (HSCL-25) was used to detect symptoms of anxiety and depression. With a reference period of the past month and composed of a 10-item anxiety subscale and a 15-item depression subscale, each item is scored on a Likert scale from 1 (not at all) to 4 (extremely). The instrument has been used as both a self-report inventory as well as an interviewer-administered scale for non-literate populations. The HSCL-25 is a frequently used scale for detecting cases of anxiety and depression and has been used in Western populations [29] and in cross-cultural research [30,31]. The researchers employed a version of the HSCL-25 previously translated into French and used in Eastern DRC [32]. In this sample, the Cronbach’s alpha was 0.86 for anxiety and 0.85 for depression. Mean symptom scores for anxiety and depression were calculated for each participant.

The 16-item version of section four of the Harvard Trauma Questionnaire (HTQ) was used to evaluate for post-traumatic stress disorder-related symptoms in the last seven days. Post-traumatic stress disorder (PTSD) syndrome is composed of a constellation of symptoms, including frequent intrusive memories of a traumatic event, avoidance of its reminders, emotional blunting, and frequent hyperarousal [33]. Refugees and similar populations who experience conflict, natural disasters, and other complex humanitarian emergencies are at a high risk of developing PTSD and associated disability and dysfunction [34]. The HTQ is one of the most frequently used screening instruments for trauma symptoms in clinical and research work with refugee populations [35]. Similar to the HSCL-25, participants respond to each question on the HTQ using a 4-point Likert scale, ranging from 1 (not at all) to 4 (a lot). For this sample, Cronbach’s alpha was 0.89. A mean symptom score was calculated for each participant.

### 2.3. Statistical Analyses

Classification of participants into patterns of gender attitudes was performed using LCA. LCA identifies unobservable characteristics—latent classes—in participants based on observable variables. Latent classes cannot be directly measured and typically represent complex constructs (e.g., happiness or behavior patterns). A participant’s response pattern to a set of observable variables generates a probability of belonging to each of the latent classes. Participants are classified into the latent class to which they have the highest likelihood of belonging. LCA has been called a person-centered approach to psychosocial measurement for its ability to group individuals who exhibit similar response patterns and not assume all individuals follow the same pattern [36].

LCA was performed using the generalized structural equation model. Using responses to the eight gender attitude items related to the acceptability of husband’s IPV and gender equality, we first estimated models with 2, 3, and 4 classes. We examined Akaike’s information criterion (AIC) and Bayesian information criterion (BIC) information statistics for each model to aid in final model selection. Smaller AICs and BICs are more desirable and represent better fit and more parsimonious models. Model selection was also done in consultation with experts in gender attitudes in Eastern DRC (authors NG, MMR, and NP), in order to select the number of classes that most appropriately fit the data.

Pearson’s chi-square test and analysis of variance were used to compare differences in demographic characteristics between classes within the final model. Simple and multivariable logistic regression was used to estimate the unadjusted and adjusted odds ratios of experiencing or perpetrating different forms of IPV between a reference class and the remaining classes within the final latent class model. Differences between the classes in the final model on anxiety, depression, and PTSD were tested with adjusted and unadjusted linear regression. Statistical significance was set at an α level ≤ 0.05. All analyses were conducted in Stata 15 [37]. Ethical approval was obtained from the Johns Hopkins Medicine Institutional Review Board.

## 3. Results

Table 1 provides demographics, experiences with IPV, and mean anxiety, depression, and PTSD scores for the overall study sample. The majority of the sample was female and married. A large portion of the sample were 45 years or older and experienced occasional food insecurity. Most participants reported experiencing or perpetrating controlling behaviors and approximately one-quarter reported experiencing or perpetrating at least one form of psychological abuse and/or, physical, or sexual violence.

Information criteria were calculated for 2-, 3-, and 4-class models. AIC and BIC values had sizeable decreases between the 2- and 3-class models (2-class: AIC 5408.969 and BIC 5488.264; and 3-class: AIC 5345.608 and BIC 5466.883). Only a marginal decrease in the AIC was observed between the 3- and 4-class models (4-class: AIC 5344.955; BIC 5508.209), while an increase in the BIC was noted. Based on low AIC and BIC values and interpretability of the classes, three classes were selected. Table 2 provides the three-class model class sample sizes and the percent who of participants who agreed with each item statement by class.

The first class was made up 13% of the overall sample and titled Tolerant of IPV, and men and women in this class were more likely to disagree with statements related to the inappropriateness of husbands’ use of IPV against their wives. The majority of participants in the second class, comprising 39% of all participants and titled Gender Equitable except Tolerant of Husband’s Home-Life Dominance, expressed agreement with statements related to the inappropriateness of husbands’ use of physical violence against their wives. While a majority of members of this class broadly agreed with statements advocating for gender equality, they were also likely to report believing that women should not participate equally with their husbands in making decisions about the household and that a wife’s opinions should not be equal to her husband’s opinions. Finally, the third class, composed of the remaining 48% of all participants, was named Fully Gender Equitable. Participants in this class expressed agreement with statements regarding the inappropriateness of husbands’ use of IPV and the vast majority endorsed statements related to the importance of gender equality.

Table 3 examines differences in the three classes on participant demographics and experiences with or perpetration of different forms of abuse. The classes differed significantly by marital status and on mean household food insecurity scores. A greater proportion of those in Fully Gender Equitable class reported being currently married than in either of the other two groups. Members of Tolerant of IPV had a higher mean household food insecurity score, indicating more frequent experiences with food insecurity and, possibly, lower economic security. The classes did not differ significantly on sex or age.

Table 4 disaggregates by latent class the frequency of ever having had experienced or perpetrated any of the different forms of IPV assessed and mean scores (range 1–4) of participants’ reported anxiety, depression, and PTSD. Using simple and multivariable logistic and regression, Table 5 compares the unadjusted and adjusted odds of experiencing or perpetrating different forms of IPV by class membership. Table 5 also explores associations between class assignment and mean scores for measures of anxiety, depression, and PTSD through simple and multivariable linear regression. For all analyses Tolerant of IPV serves as the reference group with adjusted models controlling for marital status and household food insecurity status.

In this sample, those classified as belonging to Gender Equitable except Tolerant of Husband’s Home-Life Dominance were nearly half as likely as those in the Tolerant of IPV class to have reported experiencing or perpetrating psychological abuse, even after adjusting for marital status and food insecurity (aOR = 0.59, *p*-value = 0.048). This same association was found to be statistically significant in the adjusted model comparing the Fully Gender Equitable class with Tolerant of IPV (aOR 0.59, *p*-value = 0.041). Additionally, those in the Gender Equitable except Tolerant of Husband’s Home-Life Dominance class have a lower odds of experienced or perpetrated physical or sexual violence than the Tolerant of IPV class (aOR 0.59, *p*-value = 0.046). There were no differences among the three classes in experiencing/perpetrating controlling behaviors.

In unadjusted models, membership in the Fully Gender Equitable class was associated with statistically significantly lower scores on measures of anxiety (β = −0.15, *p*-value = 0.014) and depression (β = −0.12, *p*-value = 0.022) than the Tolerant of IPV class. These associations, however, were not significant in adjusted models. Membership in the Gender Equitable except Tolerant of Husband’s Home-Life Dominance class was associated with significantly lower PTSD scores in both the unadjusted and adjusted models (β = −0.25, *p*-value < 0.001), when compared to the Tolerant of IPV class. Similarly, membership in the Fully Gender Equitable class was associated with significantly lower PTSD scores in both the unadjusted and adjusted models ((β = −0.34, *p*-value < 0.001), when compared to the Tolerant of IPV class.

## 4. Discussion

In this study, we were able to classify participants based on their gender attitudes and measure associations between these attitudes and histories of experiencing or perpetrating IPV and current mental health problems. The analysis identified three classes, Tolerant of IPV, Gender Equitable except Tolerant of Husband’s Home-Life Dominance and Fully Gender Equitable. The distribution of individuals within these three classes indicates that, despite living in a post-conflict, cultural context favoring gender attitudes of hegemonic masculinity, nearly half of all participants expressed preferences that classified them as belonging to the Fully Gender Equitable class, a group opposed to men’s use of IPV and favorable of gender equality. This opposition to IPV is higher than previous scholarship conducted in the DRC in which approximately two-thirds of male and female participants reported believing that husbands’ physical punishment of their spouses was acceptable in at least some circumstances [38]. Despite largely condoning husbands’ physical punishment against their spouses, members of Tolerant of IPV agreed in equal proportion to members of Gender Equitable except Tolerant of Husband’s Home-Life Dominance that it was important to send both daughters and sons to school and in similar proportions with both the other classes that daughters should not be married before 15 years age. Additionally, members of Tolerant of IPV reported greater agreement with statements relating to the importance of equal decision-making in the home and supporting women in leadership roles than members of Gender Equitable except Tolerant of Husband’s Home-Life Dominance.

Attitudes of agreement and disagreement across different gender equitable behaviors highlight the multidimensional, rather than unitary, nature of gender equality as a construct, and suggest that participants’ level of acceptance or rejection of gender equality does not exist along a single continuum. For example, 83% of those classified as Tolerant of IPV while only 40% of those classified as Gender Equitable except Tolerant of Husband’s Home-Life Dominance agreed with the statement, “Women should participate equally with their husbands in making decisions about the household.” Individuals’ gender equality attitudes are contextually dependent and sometimes inconsistent. These incongruities in attitudes reflect complex balances of power between men and women and diverse manifestations of patriarchy [39]. As Glick and Fisk (1996) observe through the Ambivalent Sexism Theory, hegemonic masculinities and inequitable gender attitudes can be operationalized through hostile or benevolent forms of sexism. While hostile sexism refers to overt attempts to subordinate women in order to preserve men’s power, benevolent sexism seeks to “protect” women’s purity and beauty. This more socially acceptable form of sexism emphasizes the complementarity of men and women and has demonstrated greater acceptance across sexes than hostile sexism [40]. These overlapping yet distinct forms of sexism could help to explain unexpected levels item agreement and disagreement in the Tolerant of IPV and Gender Equitable except Tolerant of Husband’s Home-Life Dominance classes.

An area of agreement across all three classes, however, was the importance of girls attending school and of delaying their marriage until after age 15. Increasing girls’ educational opportunity and delaying marriage until after adolescence are known to have many beneficial effects on individual and community economic development and on the health and well-being of girls and their future children [41,42,43,44,45]. Participants within this region of the DRC have been exposed to multiple years of humanitarian and development programing that has included messaging by government and NGO authorities regarding the benefits of educating girls and avoiding forced/early marriage and see adopting these behaviors as critical to improving family socioeconomic mobility. Broad acceptance of statements related to girls’ education and avoidance of adolescent marriage, regardless of other gendered beliefs, becomes a pragmatic decision once parents appreciate how it can enhance family health and wealth.

While a significant portion of the sample expressed attitudes intolerant of IPV, numerous participants in the overall sample reported ever experiencing or perpetrating acts of IPV. Over 60% of participants reported experiences with controlling behaviors, 25% with psychological abuse, and 28% with physical and/or sexual violence. These rates are similar to those reported by sample of Tanzanian adolescent and young women but lower than other samples of conflict-affected adolescent girls and women in South Sudan and the DRC [46,47,48]. Attitudes towards men’s perpetration of violence against women is a factor known to influence IPV’s acceptability, its overall incidence rate, and the responses of survivors and those close to them [49]. Flynn and Graham (2010) have previously conceptualized such gendered attitudes as a stable personality trait that, when interacting with more proximal factors (e.g., stressful life circumstances, desires to assert power or authority) lead to increased risks for IPV [50].

In this study, membership to Gender Equitable except Tolerant of Husband’s Home-Life Dominance or Fully Gender Equitable was associated with lower odds of experiencing or perpetrating psychological abuse in adjusted models. Further, membership to Gender Equitable except Tolerant of Husband’s Home-Life Dominance, a class expressing gender equitable attitudes but accepting of men’s primacy within the domestic sphere, had nearly half the odds of reporting ever experiencing or perpetrating physical and/or sexual violence as compared to those in the reference group, Tolerant of IPV. While more gender equitable attitudes were found to be associated with lower odds of ever experiencing or perpetrating violence, these results should not be interpreted as a causal relationship beginning with a woman’s gender inequitable attitudes and leading to a higher likelihood of being a victim of psychological or physical abuse of sexual violence. To do so would be to blame the victim.

Within the study of IPV, status inconsistency theory has been used to explain how women in gender inequitable, low-resource households are at increased risk for IPV due to relationship strain related to these resource constraints and limited alternatives for safety. The theory also argues that women in high status and resource households might be at similar risks for IPV because of perceived challenges to social norms around male superiority. The risk for IPV is actually the lowest for women in households that have sufficient resources but whose status does not threaten traditional patriarchal practices [51]. The U-shaped decrease in odds of reporting physical and/or sexual violence perpetration in Gender Equitable except Tolerant of Husband’s Home-Life Dominance or Fully Gender Equitable could be evidence in support of the status inconsistency theory [52].

These findings support existing scholarship that indicates that endorsement of hegemonic masculine and tolerance of violence attitudes are associated with increased odds of IPV perpetration and victimization [53,54]. Within the classes that endorse gender inequitable attitudes, such as the Tolerant of IPV class, IPV has been described as a tool to punish female spouse behavior that contravenes strict gender roles. Women’s endorsement of attitudes tolerant of IPV is the result of socialization within home environments that support men’s right to use IPV as a form of discipline [38]. This use of IPV by men against their spouses is not only an enormous threat to the mental and physical health of women globally, but also an influential factor in the future attitudes and behaviors of children who witness it [55,56]. Children who observe violence against their mothers or are victims of it themselves internalize the lesson that violence is an acceptable conflict resolution tool and may go on to model these behaviors later in life [57].

Associations between class membership and mental health outcomes were also detected. Within unadjusted models, those in the Fully Gender Equitable class had statistically significantly lower symptoms of anxiety, depression, and PTSD scores. However, differences between classes on measures of symptoms of anxiety and depression were no longer found to be significant after controlling for marital status and food insecurity (i.e., socioeconomic status). Membership in either Gender Equitable except Tolerant of Husband’s Home-Life Dominance or Fully Gender Equitable was associated with lower mean PTSD symptom scores in both unadjusted adjusted models, however. Masculinity-related constructs have been previously positively associated with negative mental health outcomes across numerous samples [58,59]. In men, this is frequently attributed to gender role conflicts that restrict emotionality and discourage help-seeking. Rather than being taught as boys to deal productively with upsetting emotions arising from traumatic experiences by expressing and exploring their feelings, men who endorse traditional, restrictive gender roles are socialized to respond outwardly. These physical responses, as our IPV analyses support, include more frequent perpetration of IPV than by those men who demonstrate weaker adherence to strict gender roles [60]. While men experience greater lifetime prevalence of traumatic events, women exhibit higher rates of PTSD [61]. Arguments have been made that differences are the result of a biological predisposition in females of experiencing more severe psychological reactions to such events [62]. However, a systematic undercounting of GBV as a key trigger for PTSD is perhaps a more compelling explanation for the disparity in rates as GBV is more strongly associated with adverse social and personal repercussions, such as self-blame, stigma, and guilt, than are other forms of violence [11,61,63,64]. The relationship between significantly lower PTSD scores and class membership to either Gender Equitable except Tolerant of Husband’s Home-Life Dominance or Fully Gender Equitable than as compared to membership to Tolerant of IPV is likely conceptually complex but could in part be attributed to lower odds of enduring traumatic IPV experiences.

## 5. Implications

Our results highlight important associations between individuals’ gender attitudes and experiences with IPV and mental health. This research supports previous scholarship arguing that restrictive gender norms negatively affect the health trajectories of both women and men [3]. Research is beginning to give a clearer picture of when gender attitudes become salient constructs in the minds of young people and the methods by which gendered behaviors are passed down from one generation to the next [5,7,8]. More research is needed to clarify the complex relationship between restrictive gender attitudes and poorer mental health. This research should aim to consider the entire family by examining parent/child dyads in order discover how such restrictive attitudes affect mental health during adolescence, a developmentally sensitive period of life.

Gender transformative interventions have been developed to help communities confront the harmful effects of restrictive gender attitudes and norms [65,66,67]. Such transformations are slow to occur and take patience and long-term commitment. Changes in gender attitudes are possible, though. Within this study, a majority of participants in all three classes reported believing that it was equally important to send girls to school as it was boys. Families seem to be acting on these beliefs. Female enrollment in secondary school (ages 12–17) in the DRC has also steadily increased, with most recent figures indicating a female secondary enrollment rate increase from approximately 30% in 2010 to nearly 36% in 2015 [68]. Ongoing inquiry is needed to inform the development of future gender transformative programs designed to help communities confront the damaging consequences of gender inequality and IPV by discovering how to more effectively engage men and boys as agents of change [69].

## 6. Strengths and Limitations

The primary strength of our analysis is its use of LCA to assign participants to different gender attitudes classes. As previously mentioned, gender equality is a complex, multifaceted construct that is difficult to accurately represent along a single-domain continuum. Using a person-centered approach to class membership identification allowed us to consider and integrate the various attitudes participants hold into our final model solution.

The following limitations should be considered when interpreting the results of this present study. Due to the cross-sectional nature of the data, it is not possible to infer a direction of causation between gender attitudes and ever experiencing or perpetrating IPV and symptoms of anxiety, depression, or PTSD. Second, social desirability bias in self-reported gender attitudes, experiences with IPV, or mental health measures could drive some findings. Third, as a secondary data analysis, the gender attitudes independent variable used in this study was not purposely designed for these analyses. As such, adaptation of the original questionnaire to meet this study’s requirements could have eliminated some response pattern nuances. Finally, as only a small segment of the study population was male, it was not possible to stratify by sex.

## 7. Conclusions

Within this study, significant associations were detected between gender attitudes, IPV and mental health. This research contributes to the expanding body of literature supporting the notion that gender attitudes exert a strong influence on physical and emotional health. Addressing gender inequitable attitudes and their negative health effects will take commitment. However, devoting time and resources to ensuring that gender equality is addressed in all global health programming will yield future dividends and enhance human development and well-being.

## Figures and Tables

**Table 1:** Demographic characteristics of sample (*n* = 784).

	Total No. (%) or Mean (SD)	Missing (%)
**Participant sex**		
Number female	674 (86%)	0 (0%)
**Age**		
20–34	241 (31%)	
35–44	201 (26%)	
45+	342 (43%)	0 (0%)
**Marital status**		
Married	543 (69%)	
Not currently married	241 (31%)	0 (0%)
**Household Food Insecurity Access Scale**		
Mean score (range 0–3)	1.38 (0.65)	0 (0%)
**Experienced or perpetrated controlling behaviors**		
Yes	478 (61%)	12 (2%)
**Experienced or perpetrated psychological abuse**		
Yes	200 (25%)	12 (2%)
**Experienced or perpetrated physical or sexual abuse**		
Yes	218 (28%)	12 (2%)
**Anxiety**		
Mean score (range 1–4)	1.64 (0.55)	0 (0%)
**Depression**		
Mean score (range 1–4)	1.62 (0.46)	0 (0%)
**PTSD**		
Mean score (range 1–4)	1.77 (0.56)	0 (0%)

**Table 2:** Latent classes and percent agreement.

	Tolerant of IPV	Gender Equitable Except Tolerant of Husband’s Home-Life Dominance	Fully Gender Equitable
	*n* = 103	*n =* 307	*n* = 374
**Husbands’ use of intimate partner violence (IPV)**			
It is not okay for a husband to beat his wife to discipline her	49%	90%	99%
A man does not have the right to beat/punish his wife	14%	93%	97%
**Gender equality**			
It is not more important to send sons to school than send a daughter to school	88%	88%	97%
Women should participate equally with their husbands in making decisions about the household	83%	40%	96%
It is not acceptable for girls to be married before 15 years of age	93%	89%	97%
Men are not more important than women in the family	58%	69%	92%
A wife should express her opinion when she disagrees with what her husband is saying	74%	51%	85%
Men should support women in leadership roles in the community	85%	76%	92%

**Table 3:** Comparison of demographic characteristics by latent class.

	Tolerant of IPV	Gender Equitable Except Tolerant of Husband’s Home-Life Dominance	Fully Gender Equitable	Χ^2^ or ANOVA *p*-Value
	*n* = 103	*n* = 307	*n* = 374	
**Participant sex**				0.092
number female	88 (85%)	274 (89%)	312 (83%)	
**Age**				
20–34	23 (22%)	98 (32%)	120 (32%)	0.062
35–44	29 (28%)	66 (21%)	106 (28%)	
45+	51 (50%)	143 (47%)	148 (39%)	
**Marital status**				
Married	71 (69%)	194 (63%)	278 (74%)	0.007 *
Not currently married	32 (31%)	113 (37%)	96 (26%)	
**Household Food Insecurity Access Scale** (range 0–3)				
Mean score (*SD*)	1.54 (0.50)	1.38 (0.71)	1.33 (0.63)	0.018 *

* *p*-value ≤ 0.05.

**Table 4:** Prevalence of ever-experienced IPV and mean mental health outcomes scores by latent class.

Latent Class	*n*	Controlling Behaviors Yes (*%*)	Psychological Abuse Yes (*%*)	Physical or Sexual Violence Yes (*%*)
**Tolerant of IPV**	99	66 (67%)	34 (34%)	35 (35%)
**Gender Equitable except Tolerant of Husband’s Home-Life Dominance**	306	180 (59%)	71 (23%)	73 (24%)
**Fully Gender Equitable**	367	232 (63%)	95 (26%)	110 (30%)
**Latent Class**	***n***	**Anxiety *M*(*SD*)**	**Depression *M*(*SD*)**	**PTSD *M*(*SD*)**
**Tolerant of IPV**	103	1.72 (0.55)	1.7 (0.43)	2.04 (0.61)
**Gender Equitable except Tolerant of Husband’s Home-Life Dominance**	307	1.7 (0.59)	1.66 (0.48)	1.75 (0.58)
**Fully Gender Equitable**	374	1.57 (0.51)	1.57 (0.46)	1.7 (0.51)

Abbreviations: n, class sample; %, percentage of class; M, mean; SD, standard deviation.

**Table 5:** Unadjusted and adjusted odds ratios (*n* = 772) and linear regressions (*n* = 784) with Tolerant of IPV as the reference group.

	Gender Equitable Except Tolerant of Husband’s Home-Life Dominance		Fully Gender Equitable	
Ever-Experience IPV Outcome	OR (95% CI)	aOR (95% CI) ^†^	OR (95% CI)	aOR (95% CI) ^†^
**Experienced or perpetrated controlling behaviors**	0.71	0.68	0.86	0.53
	(0.44 to 1.15)	(0.35 to 1.31)	(0.54 to 1.37)	(0.27 to 1.01)
**Experienced or perpetrated psychological abuse**	0.58	0.59	0.67	0.59
	(0.35 to 0.95) *	(0.35 to 0.99) *	(0.41 to 1.07)	(0.35 to 0.98) *
**Experienced or perpetrated physical or sexual violence**	0.57	0.59	0.78	0.70
	(0.35 to 0.93) *	(0.35 to 0.99) *	(0.49 to 1.25)	(0.43 to 1.15)
**Mental Health Outcome**	**β (95% CI)**	**aβ (95% CI) ^†^**	**β (95% CI)**	**aβ (95% CI) ^†^**
**Anxiety**	−0.02	0.01	−0.15	−0.09
	(−0.14 to 0.1)	(−0.1 to 0.12)	(−0.27 to −0.03) *	(−0.2 to 0.02)
**Depression**	−0.03	−0.001	−0.12	−0.05
	(−0.14 to 0.07)	(−0.09 to 0.09)	(−0.22 to −0.02) *	(−0.14 to 0.04)
**PTSD**	−0.29	−0.25	−0.34	−0.27
	(−0.41 to −0.17) *	(−0.36 to −0.13) *	(−0.46 to −0.21) *	(−0.38 to −0.16) *

Abbreviations: IPV, intimate partner violence; OR odds ratio; β, slope coefficient. ^†^ The adjusted regression controlled for participant marital status and household food insecurity status. * *p*-value ≤ 0.05.
